# Glutathione trisulfide prevents lipopolysaccharide-induced retinal inflammation via inhibition of proinflammatory cytokine production in glial cells

**DOI:** 10.1038/s41598-023-38696-4

**Published:** 2023-07-17

**Authors:** Hiroshi Tawarayama, Kota Umeki, Maki Inoue-Yanagimachi, Naoki Takahashi, Hirokazu Hasegawa, Noriko Himori, Satoru Tsuda, Hiroshi Kunikata, Takaaki Akaike, Toru Nakazawa

**Affiliations:** 1 Department of Ophthalmology, Tohoku University Graduate School of Medicine, 2-1 Seiryo-machi, Aoba-ku, Sendai, 980-8575 Japan; 2 Department of Retinal Disease Control, Tohoku University Graduate School of Medicine, Sendai, 980-8574 Japan; 3 Department of Aging Vision Healthcare, Tohoku University Graduate School of Biomedical Engineering, Sendai, 980-8579 Japan; 4 Department of Environmental Medicine and Molecular Toxicology, Tohoku University Graduate School of Medicine, Sendai, 980-8575 Japan; 5 Collaborative Program of Ophthalmic Drug Discovery, Tohoku University Graduate School of Medicine, Sendai, 980-8574 Japan; 6 Department of Advanced Ophthalmic Medicine, Tohoku University Graduate School of Medicine, Sendai, 980-8574 Japan

**Keywords:** Microglia, Retina, Interleukins, Acute inflammation

## Abstract

We aimed to investigate the impact of glutathione trisulfide (GSSSG) on lipopolysaccharide (LPS)-induced inflammation in retinal glia. Inflammatory responses in mouse-derived glial cells and Wistar rat retinas were stimulated with administration of LPS. Cell survival and proinflammatory cytokine production were examined using the Calcein-AM assay, and reverse transcription-quantitative polymerase chain reaction (RT-qPCR) and enzyme-linked immunosorbent assay (ELISA), respectively. Retinal microglia were visualized with immunohistochemistry for Iba1. Administration of LPS (10 µg/mL) or GSSSG (less than 100 µM) did not affect survival of cultured primary Müller cells and established microglial cells (BV-2). RT-qPCR and ELISA indicated that GSSSG inhibited LPS-induced gene upregulation and protein secretion of proinflammatory cytokines in these glial cells and rat retinas. GSSSG inhibited LPS-induced activation of TGF-β-activated kinase 1 (TAK1), which is an upstream kinase of NF-κB, in BV-2 cells. Finally, in vivo experiments indicated that intravitreal administration of GSSSG but not its relative glutathione disulfide (GSSG) inhibited LPS (500 ng)-induced accumulation of Iba1-immunopositive microglia in rat retinas. Taken together, GSSSG has the potential to prevent pathogenesis of inflammation-associated ocular diseases by inhibiting proinflammatory cytokine expression in retinal glial cells.

## Introduction

Many cells respond to injury by secreting proinflammatory cytokines and chemokines, such as tumor necrosis factor-alpha (TNF-α), interleukin (IL)-1β, IL-6, and C–C motif chemokine ligand 2 (Ccl2)^1–6^. TNF-α and IL-1β are produced in the early stage of inflammation and upregulate expression of themselves and other cytokines including IL-6^7–12^. IL-1β and IL-6 work synergistically to activate macrophages and B- and T-cells^13–17^. Ccl2 recruits monocytes and lymphocytes to inflammation sites via chemotaxis^18,19^. These molecular and cellular events lead to a severe inflammatory state in a broad range of tissues, causing irreversible damage.

Retinal glial cells, including Müller cells and microglia, play crucial roles in maintaining retinal structure, homeostasis, and nutrition^20,21^. Müller cells are prominent glia that interact with the majority of retinal cell types and have the ability to produce inflammatory mediators^22–26^. Microglia are resident macrophage-like cells that release inflammatory mediators and reactive oxygen species (ROS) in response to harmful stimuli^27^. Past studies indicated that dysregulation of these mediators in glial cells contributed to the pathogenesis of retinal degeneration diseases in human patients and retinal damage in experimental animal models^20,28,29^. Thus, glial cells are a potential therapeutic target to prevent inflammation-related retinal degeneration diseases and to attenuate the symptoms.

Glutathione trisulfide (GSSSG) belongs to a group of reactive sulfane sulfur species and is found endogenously in animal- and human-derived samples^30–32^. Glutathione polysulfides, mainly consisting of GSSSG, effectively quench ROS in the presence of glutathione disulfide reductase, which converts the oxidized form of glutathione into the reduced form^31^. GSSSG displays stronger inhibitory effects on oxidative stress-induced cell death in vitro compared to its relative, GSSG^30,31^. Furthermore, a recent in vitro study revealed a novel function of GSSSG, i.e., inhibition of stimulant-induced proinflammatory gene upregulation in a retinal pigment epithelial cell line established from human eyes^33^. However, it remains elusive whether GSSSG exerts similar inhibitory effects on other retinal cell types including glial cells, which are major sources of proinflammatory cytokines in pathological conditions.

Attenuation of proinflammatory gene expression reduces the risk of retinal degeneration. In the present study, we examined the impacts of the reactive sulfur species GSSSG on LPS-induced proinflammatory responses in retinal glial cells in vitro and in vivo.

## Methods

### Animals

C57BL/6J mice and Wistar rats (8–10 weeks old) were purchased from Japan SLC (Shizuoka, Japan) and maintained at animal facilities in Tohoku University Graduate School of Medicine (Sendai, Japan) under a 12-h light/dark cycle. Male and female mice were mated to obtain pups. All animal experiments were approved by the Committee on Animal Research at Tohoku University, and performed in agreement with the the Association for Research in Vision and Ophthalmology (ARVO) statement for the use of animals in ophthalmic and vision research and ARRIVE (Animals in Research: Reporting In Vivo Experiments) guidelines.

### Cell culture

Mouse-derived Müller cells were obtained as described previously^34–36^. Briefly, eyes dissected from postnatal day (P)5 to P8 pups were incubated in Dulbecco's modified Eagle medium (DMEM; Thermo Fisher Scientific, Waltham, MA) containing 10% fetal bovine serum (FBS; Thermo Fisher Scientific) at room temperature overnight. Retinas were isolated from eyes using sharp forceps 15 min after treatment with 0.25% trypsin–EDTA solution at 37 °C for 15 min and dissociated into small pieces by pipetting several times. The small retinal explants were cultured in DMEM containing 10% FBS in a 5% CO_2_ incubator at 37 °C. The mouse brain-derived microglial cell line BV-2 was substituted for primary retinal microglia in this study since the number of retina-derived primary microglia was expected to be small. Past studies indicated that both primary microglia and BV-2 cells possess common properties and produce the same kinds of proinflammatory cytokines^37–39^.

### Cell viability assay

Cell viability was determined using Calcein-AM (Dojindo, Kumamoto, Japan). Müller (0.5 × 10^4^ cells/well) and BV-2 cells (5 × 10^4^ cells/well) were cultured in 96-well cell culture plates in media containing various concentrations of GSSSG (Kyowa Hakko Bio, Tokyo, Japan) or LPS (Sigma-Aldrich, St. Louis, MO, USA) for 6 h. After washing with Dulbecco's phosphate-buffered saline (DPBS), cells were incubated in DPBS containing 2 µM Calcein-AM for 30 min at 37 °C. Cells were lysed with DPBS solution containing 1% Triton X-100 (FUJIFILM Wako pure chemical, Osaka, Japan), and then fluorescence intensity was measured at 515 nm (excitation: 490 nm) using a SpectraMax M2e microplate reader (Molecular Devices, San Jose, CA).

### In vitro treatment with GSSSG and LPS

Müller and BV-2 cells were maintained and expanded in 10-cm dishes containing DMEM (10% FBS) in a 5% CO_2_ incubator at 37 °C. One day before the experiments, Müller (0.5 × 10^4^ cells/well) and BV-2 cells (5 × 10^4^ cells/well) were seeded in each well of 96-well culture plates. The next day, cells were pretreated with various concentrations of GSSSG for 1 h. Subsequently, 10 µg/mL of LPS (Sigma-Aldrich) was added to the cultures, and the cells were incubated for an additional 6 hour to induce the expression of proinflammatory genes.

### Enzyme-linked immunosorbent assay (ELISA)

Concentrations of inflammation-related proteins in cell culture media were quantified using Quantikine ELISA Kit (R&D Systems, Minneapolis, MN, USA) according to the manufacturer's instructions. Briefly, glial cells were treated with GSSSG and LPS as described above. The supernatants were collected 6 h and 24 h after LPS treatment and used for ELISA-based quantification of IL-6 (6 h), and TNF-α, IL-1β, and Ccl2 (24 h).

### In vivo administration of glutathiones and LPS

Rats were anesthetized using intraperitoneal injection of 8 mg/kg xylazine (Bayer Yakuhin, Osaka, Japan) and 80 mg/kg ketamine (Daiichi Sankyo, Tokyo Japan). Rats were intravitreally administered with 2 µL of a mixture of LPS (250 ng/µL, total 500 ng/eye) and GSSSG or GSSG (7.5 or 30 nmol/µL, total 15 or 60 nmol/eye) in Ca^2+^- and Mg^2+^-free phosphate-buffered saline (PBS; Nacalei Tesque, Kyoto, Japan) using a micro-syringe with a 32G needle (Ito, Shizuoka, Japan) and then sacrificed at 10 h and 48 h post-administration to investigate proinflammatory gene expression and microglial activation, respectively. The appropriate LPS concentration to stimulate inflammatory responses was determined in our preliminary experiments.

### Quantitative reverse transcription-polymerase chain reaction (RT-qPCR)

For in vitro experiments, cell lysis and cDNA synthesis were performed using the SuperPrepII cell lysis & RT kit (Toyobo, Osaka, Japan) according to the manufacturer's instructions. For in vivo experiments, total RNA was extracted from the rat retina using the miRNeasy mini kit (QIAGEN, Hilden, Germany) and then reverse-transcribed into cDNA using the SuperScript III First-Strand Synthesis System (Thermo Fisher Scientific, Waltham, MA, USA). RT-qPCR was performed in a 7500 fast real-time PCR system (Thermo Fisher Scientific) using TaqMan fast universal PCR master mix (Thermo Fisher Scientific) and a mixture of predesigned TaqMan primers and probes (Thermo Fisher Scientific or Integrated DNA Technologies, Coralville, IA, USA) (see Supplementary Table S1).

### Immunohistochemistry

Immunostaining on whole retinas was performed following as previously described^40^. Briefly, retinas were dissected from eyes fixed with 4% paraformaldehyde in PBS for 1 h at room temperature and then postfixed with the same fixative solution overnight at 4 °C. After treating with 10% normal donkey serum in PBS containing 0.1% tween 20, retinas were incubated with antibodies for Iba1 (FUJIFILM Wako pure chemical; 019–19,741; 1:500 dilutions) for 3 days, washed, and incubated with Cy3-conjugated anti-rabbit IgG (Jackson ImmunoResearch; 711-165-152; 1:500 dilution).

### Image acquisition and quantification of Iba1-immunopositive microglia

Four fluorescent images per flat-mounted retina were captured 1 mm from the edge of the optic nerve head using a BZ-9000 fluorescence microscope with a 10 × objective lens (Keyence, Osaka, Japan). Contrast and brightness adjustment and photo trimming were performed in Adobe Photoshop Elements (Adobe Systems, San Jose, CA, USA). The number of Iba1-immunopositive microglia was counted with ImageJ software (NIH, Bethesda, MD, USA), and cell density is expressed per mm^2^. The average was calculated based on data obtained from four retinas per each experimental group.

### Western blotting

BV-2 cells were treated with LPS (10 µg/mL) and/or GSSSG (200 µM) as described above. Cells were collected and sonicated using a Bioruptor UCD-300 (Cosmo Bio, Tokyo, Japan) in RIPA buffer containing inhibitor cocktails for proteases and phosphatases (Cayman Chemical, Ann Arbor, MI, USA). Sonicated cells were centrifuged to obtain whole-cell lysates, and protein concentrations were determined using the Pierce BCA Protein Assay Kit (Thermo Fisher Scientific). Total protein (30 µg) was analyzed by western blotting according to routine protocols. Antibodies used for immunochemical experiments are listed in Supplementary Table S2. The bands on the Western blots were quantified using ImageJ software (National Institutes of Health, Bethesda, MD, USA).

### Statistical analyses

Quantitative data were analyzed using Welch's *t*-test for two experimental groups, and analysis of variance, followed by Tukey–Kramer and Dunnett's post-hoc tests, was performed for more than two experimental groups. Analyses were performed using JMP Pro 14 software (SAS Institute, Cary, NC, USA), and *P* < 0.05 was considered significant.

## Results

### Cell viability of Müller and BV-2 cells treated with GSSSG or LPS

**Figure 1:**
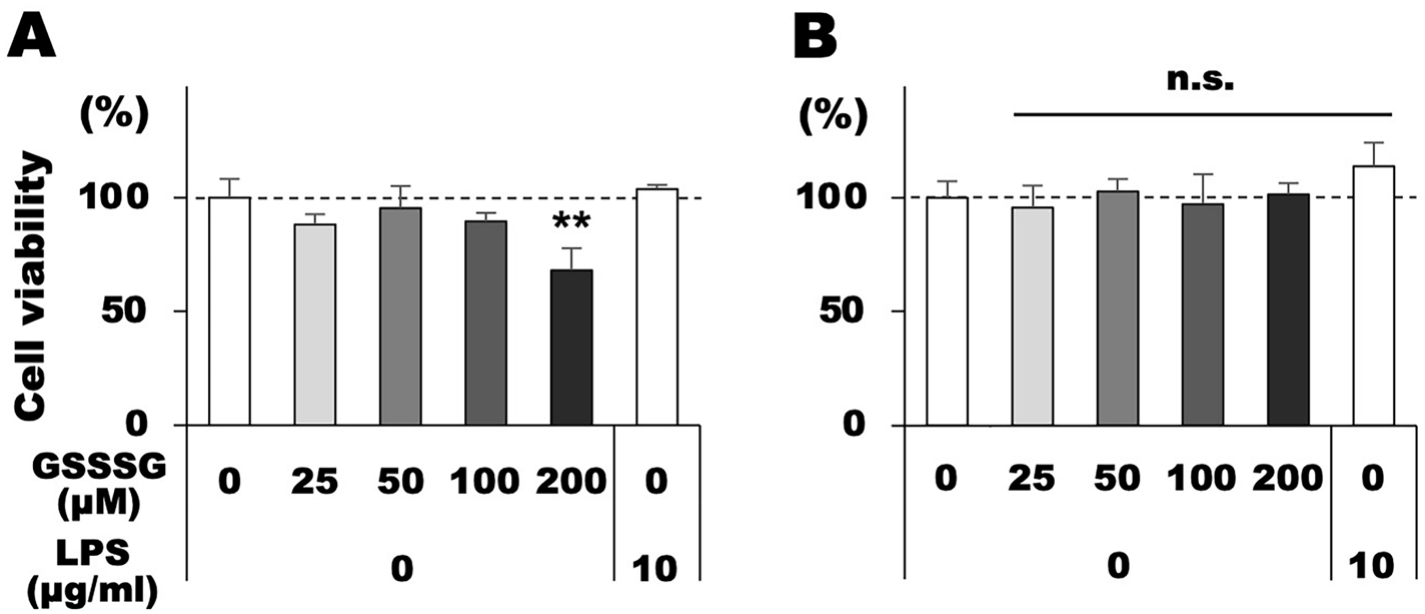
Cell survival of Müller and BV-2 cells treated with GSSSG or LPS. Cell viability of Müller (**A**) and BV-2 cells (**B**) treated with various concentrations of GSSSG or LPS for 6 h. Living cells were detected using the Calcein-AM assay, and viability is shown as a percentage of LPS( −)GSSSG( −) controls. ^**^P < 0.01 versus LPS( −)GSSSG( −) (Dunnett's test; *n* = 4).

We first investigated the toxicity of GSSSG and LPS on mouse-derived Müller and BV-2 microglial cells. Cells were treated with various concentrations of GSSSG (25–200 µM) or LPS (10 µg/mL) for 6 h, and then living cells were detected using Calcein-AM. GSSSG had no effect on the Calcein-AM signal in Müller or BV-2 cells at concentrations less than 100 µM (Fig. 1A,B). However, a higher concentration (200 µM) of GSSSG decreased the viability of Müller but not BV-2 cells (Fig. 1A,B). LPS treatment did not affect the viability of both Müller and BV-2 cells in the assay period examined (Fig. 1A,B).

### GSSSG inhibited LPS-induced upregulation of proinflammatory genes and secretion of proteins

**Figure 2:**
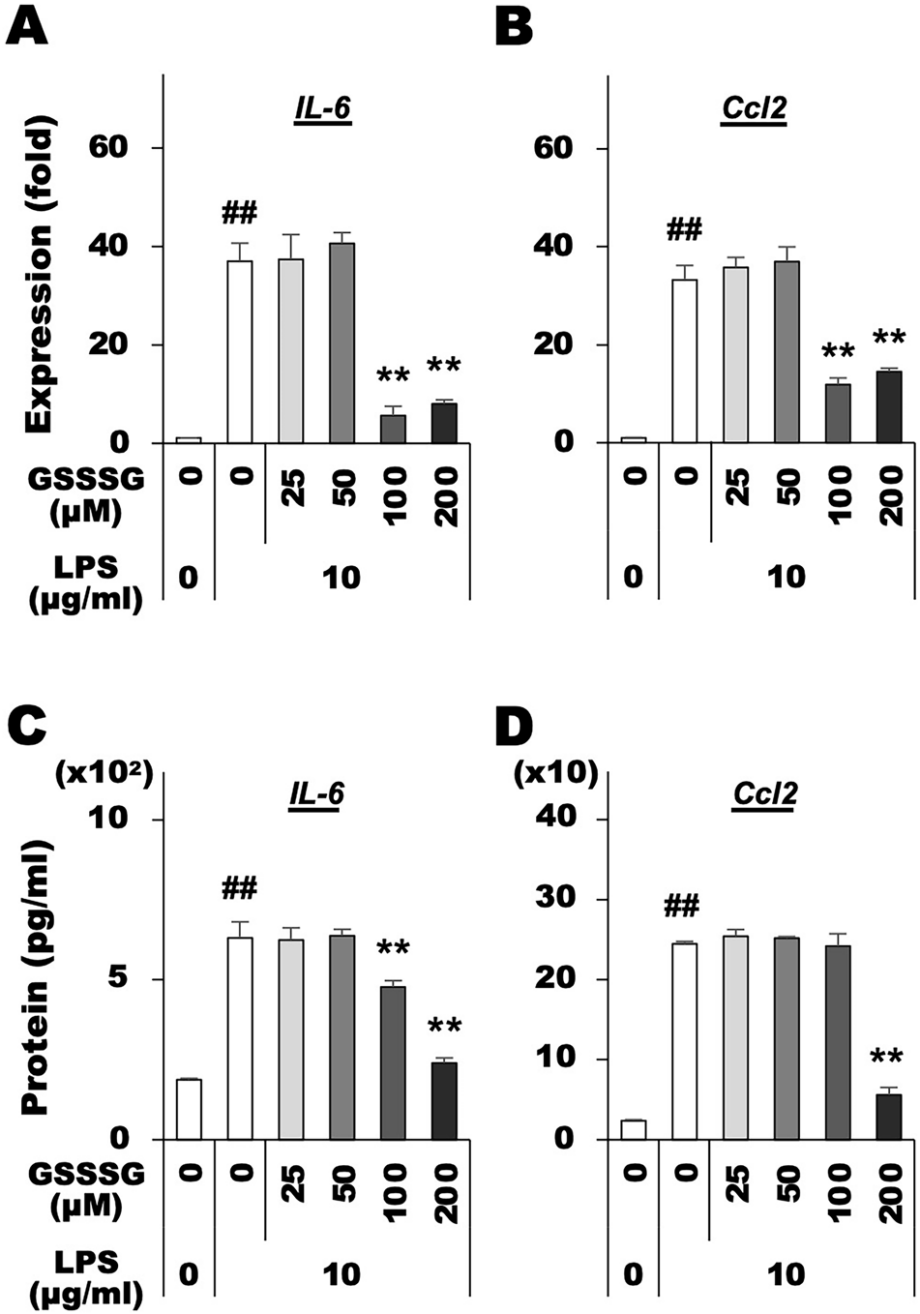
Inhibitory effects of GSSSG on LPS-induced expression of proinflammatory cytokines in Müller cells. Changes of IL-6 and Ccl2 mRNA expression (**A** and **B**) and protein secretion (**C** and **D**) in mouse primary Müller cells. For gene expression analysis using RT-qPCR (**A** and **B**), cells were pretreated with GSSSG for 1 h, followed by LPS treatment for 6 h. For protein quantification using ELISA (**C** and **D**), cells were also pretreated with GSSSG for 1 h, then subjected to LPS treatment for either 6 h or 24 h. Culture supernatants obtained from cells treated with LPS for 6 h and 24 h were used for protein quantification of IL-6 and Ccl2, respectively. Error bars represent the standard deviation of the mean. ^##^*P* < 0.01 versus LPS( −)GSSSG( −) controls (Welch's *t* test; *n* = 4); **P < 0.01 versus LPS( +)GSSSG( −) (Dunnett's test; *n* = 4).

We then examined the effects of GSSSG pretreatment on LPS-induced expression of mRNAs and proteins from proinflammatory genes in the culture media of Müller and BV-2 cells using RT-qPCR and ELISA, respectively. LPS stimulated expression of *IL-6* and *Ccl2* mRNAs (Fig. 2A,B) and secretion of their respective proteins (Fig. 2C,D) in Müller cells. However, pretreatment of Müller cells with GSSSG resulted in inhibition of the LPS-induced increase in mRNA expression and protein secretion (Fig. 2A–D).

**Figure 3:**
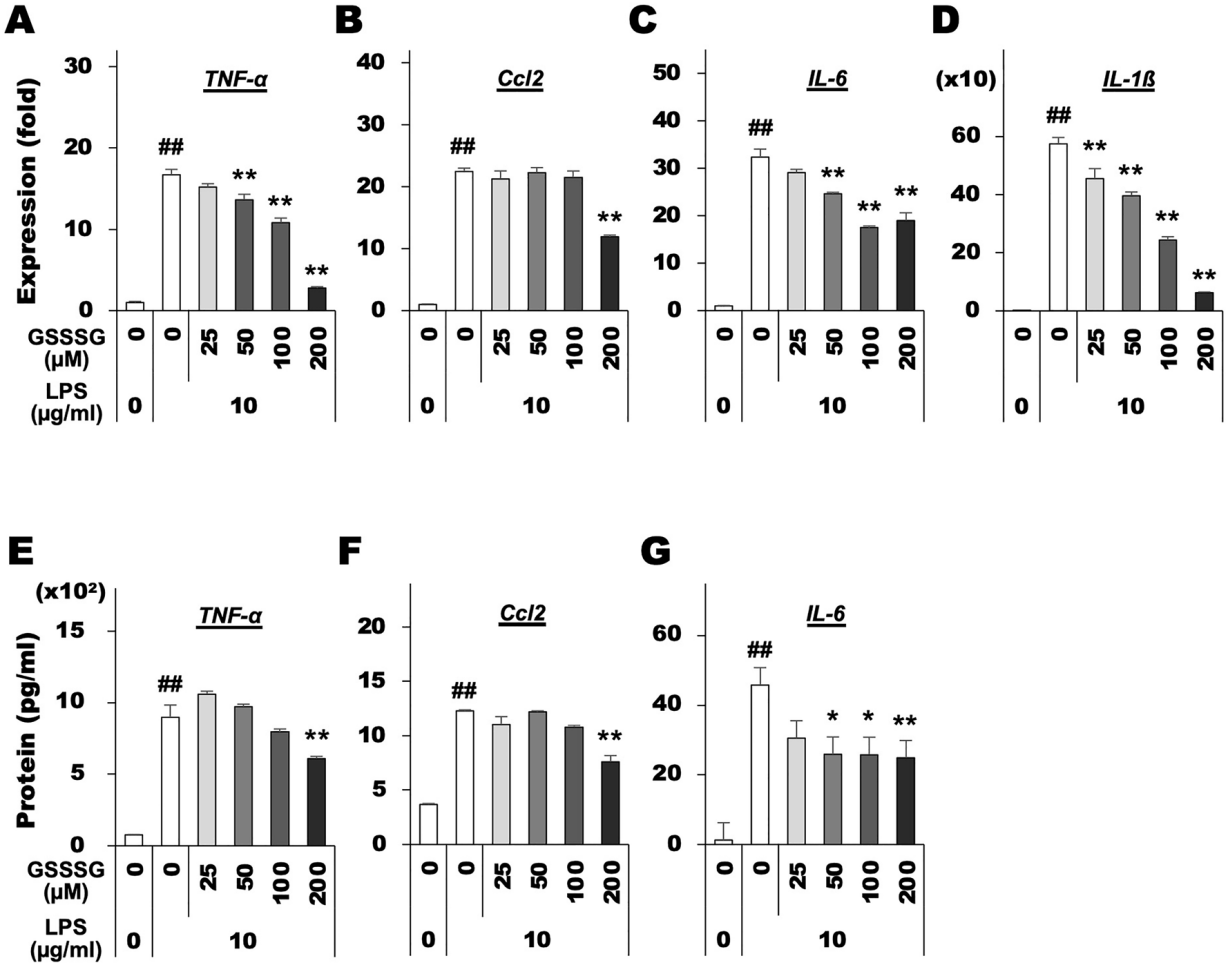
Inhibitory effects of GSSSG on LPS-induced expression of proinflammatory cytokines in BV-2 cells. Changes of TNF-α, Ccl2, IL-6 and IL-1β mRNA expression (**A–D**) and protein secretion (**E–G**) in mouse-derived BV-2 cells. For gene expression analysis using RT-qPCR (**A-D**), cells were pretreated with GSSSG for 1 h, followed by LPS treatment for 6 h. For protein quantification using ELISA (**E–G**), cells were pretreated with GSSSG for 1 h, followed by LPS treatment for 6 h or 24 h. Culture supernatants obtained from cells treated with LPS for 6 h and 24 h were used for protein quantification of IL-6 (6 h), and TNF-α, Ccl2 and IL-1β (24 h). Error bars represent the standard deviation of the mean. ^##^*P* < 0.01 versus LPS( −)GSSSG( −) controls (Welch's *t* test; *n* = 4); ^*^P < 0.05 and **P < 0.01 versus LPS( +)GSSSG( −) (Dunnett's test; *n* = 4).

Upregulated expression of proinflammatory genes was also detected in LPS-stimulated BV-2 cells. In addition to Ccl2 and IL-6, the expression levels of inflammatory mediators TNF-α and IL-1β were significantly increased in the presence of LPS (Fig. 3A–D). However, GSSSG pretreatment attenuated the LPS-induced upregulation of these inflammatory genes (Fig. 3A–D). ELISA-based protein quantification indicated that LPS stimulated secretion of proinflammatory proteins including TNF-α, Ccl2, and IL-6 from BV-2 cells (Fig. 3E–G). However, GSSSG administration attenuated the increased secretion of these proteins (Fig. 3E–G). The concentration of IL-1β protein was below the limit of detection (data not shown).

### GSSSG inhibited proinflammatory gene expression in LPS-challenged rat retinas

**Figure 4:**
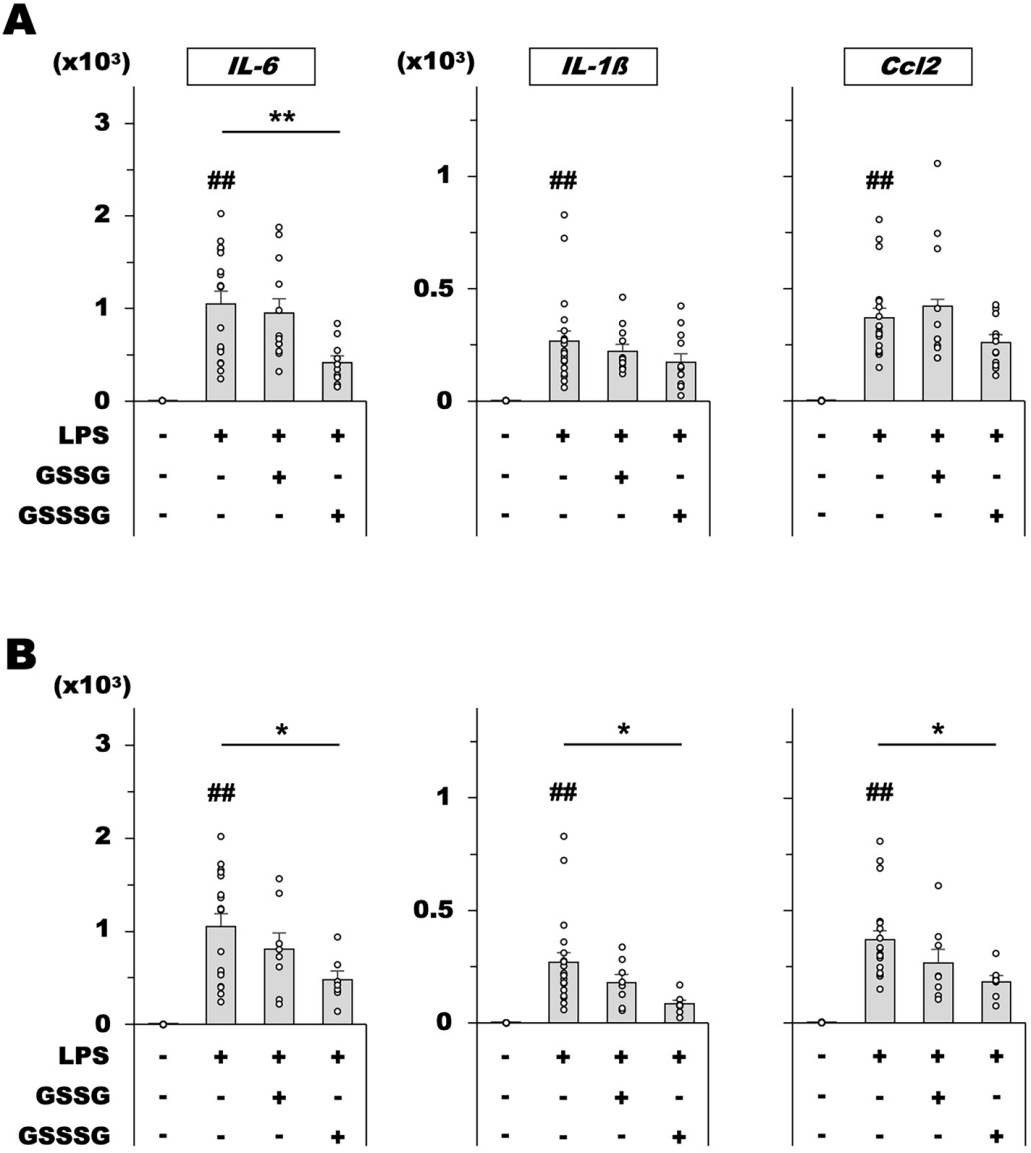
In vivo effects of GSSSG and GSSG on LPS-induced upregulation of proinflammatory cytokine genes in rat retinas. Changes in *IL-6*, *IL-1β*, and *Ccl2* mRNA levels in retinas dissected from rat eyes treated with LPS and GSSSG or GSSG. Rats were intravitreally injected with LPS (500 ng) and GSSSG or GSSG (15 or 60 nmol) simultaneously, resulting in putative final glutathione concentrations of 300 µM (**A**) and 1,200 µM (**B**) in the vitreous humor. Retinas were collected 10 h after administration. Error bars represent the standard deviation of the mean. ^##^*P* < 0.01 versus LPS ( −)GSSSG( −) controls; ^*^P < 0.05, ^**^P < 0.01 (Tukey–Kramer test; *n* = 12 − 20 in **A**, 7 − 20 in **B**. Data for the LPS( −)glutathiones( −) and LPS( +)glutathiones( −) treatment in **B** are the same as those in **A**.

We then examined whether GSSSG exerted similar anti-inflammatory activity in animal retinas as that observed in vitro. To this end, GSSSG or GSSG was intravitreally administered to rat eyes together with LPS, and changes in the expression of proinflammatory genes were analyzed at 10 h post-administration by RT-qPCR. Intravitreal injection of LPS (500 ng) upregulated *IL-6*, *IL-1β*, and *Ccl2* expression in the retina (Fig. 4A,B), whereas concomitant administration of GSSSG (15 nmol) significantly decreased the upregulation of *IL-6* but not *IL-1β*, and *Ccl2* (Fig. 4A). However, GSSSG administration with a higher amount (60 nmol) significantly attenuated *IL-6*, *IL-1β*, and *Ccl2* upregulation in LPS-challenged retinas (Fig. 4B). In contrast, GSSG administration led to no significant reduction in their expression (Fig. 4A,B).

### GSSSG attenuates microglial accumulation in LPS-challenged rat retinas

**Figure 5:**
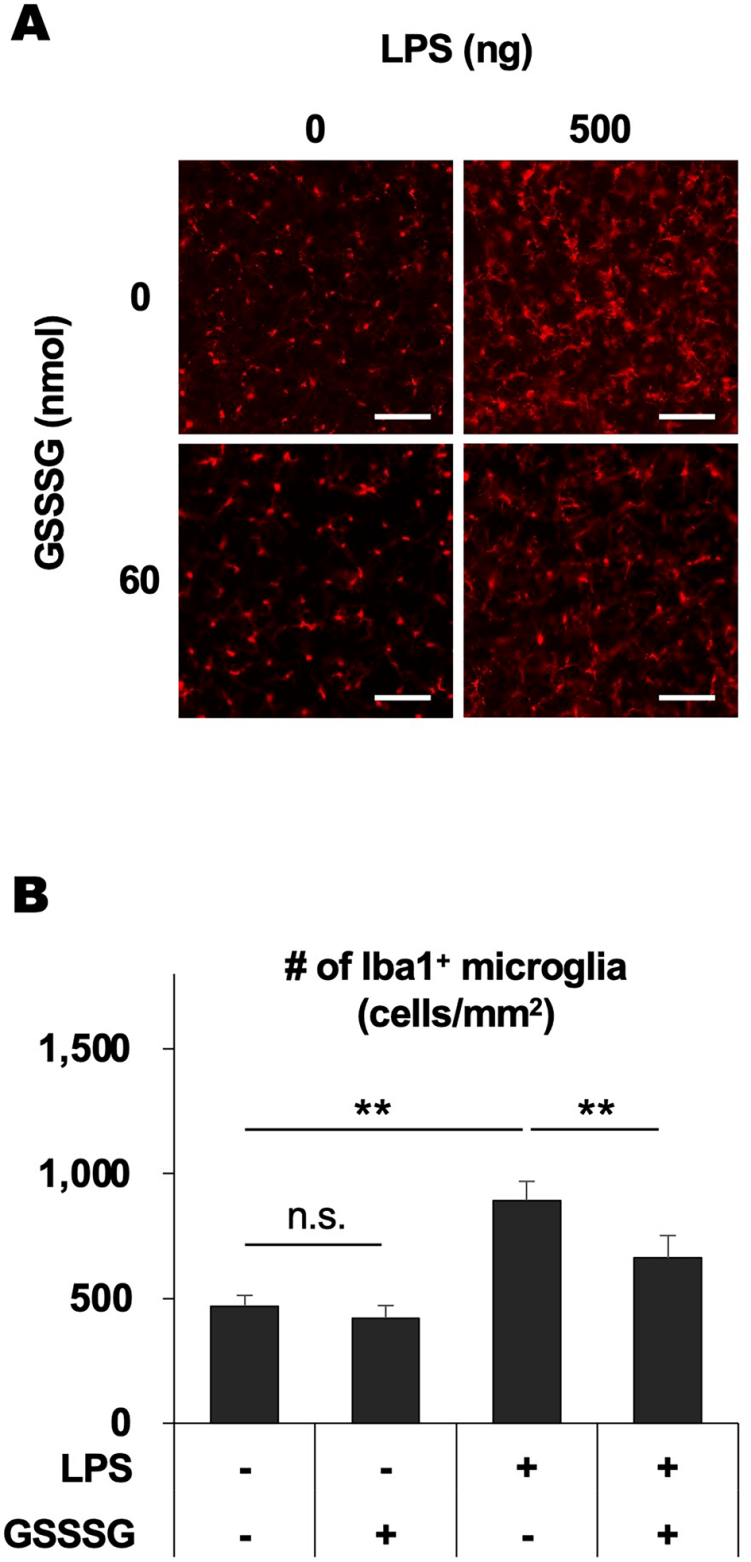
Inhibitory effects of GSSSG on LPS-induced accumulation of microglia in rat retinas. (**A**) Iba1 immunostaining of flat-mounted retinas obtained from LPS- and GSSSG-challenged rats. Rats were intravitreally injected with LPS (500 ng) and GSSSG or GSSG (60 nmol) simultaneously and sacrificed 48 h later. Scale bars: 100 µm. (**B**) Quantification of Iba1-immunopositive microglia in retinas treated with LPS and GSSSG. Error bars represent the standard deviation of the mean. **P < 0.01 (Tukey–Kramer test; *n* = 4). n.s.: not significant.

We then examined the impact of GSSSG on microglial accumulation in LPS-challenged rat retinas. Rats were intravitreally administered with LPS alone or in combination with GSSSG and sacrificed 48 h later, and then dissected retinas were immunostained for the microglial marker Iba1 (Fig. 5A). The immunohistochemical analysis of flat-mounted retinas indicated that LPS administration resulted in vigorous extension of microglial processes and a significant increase in the number of Iba1-immunopositive microglia (Fig. 5B). However, concomitant administration of GSSSG attenuated LPS-induced accumulation of microglia (Fig. 5B). Administration of GSSSG itself did not affect the number of Iba1-immunopositive microglia (Fig. 5A,B). Additionally, no drastic changes were observed in the retinal structure and expression pattern of glial cell markers in the retinas of rats received intravitreal administration of GSSSG (60 nmol) (Supplementary Fig. S1).

### GSSSG inhibits LPS-induced phosphorylation of TAK1 in BV-2 cells

**Figure 6:**
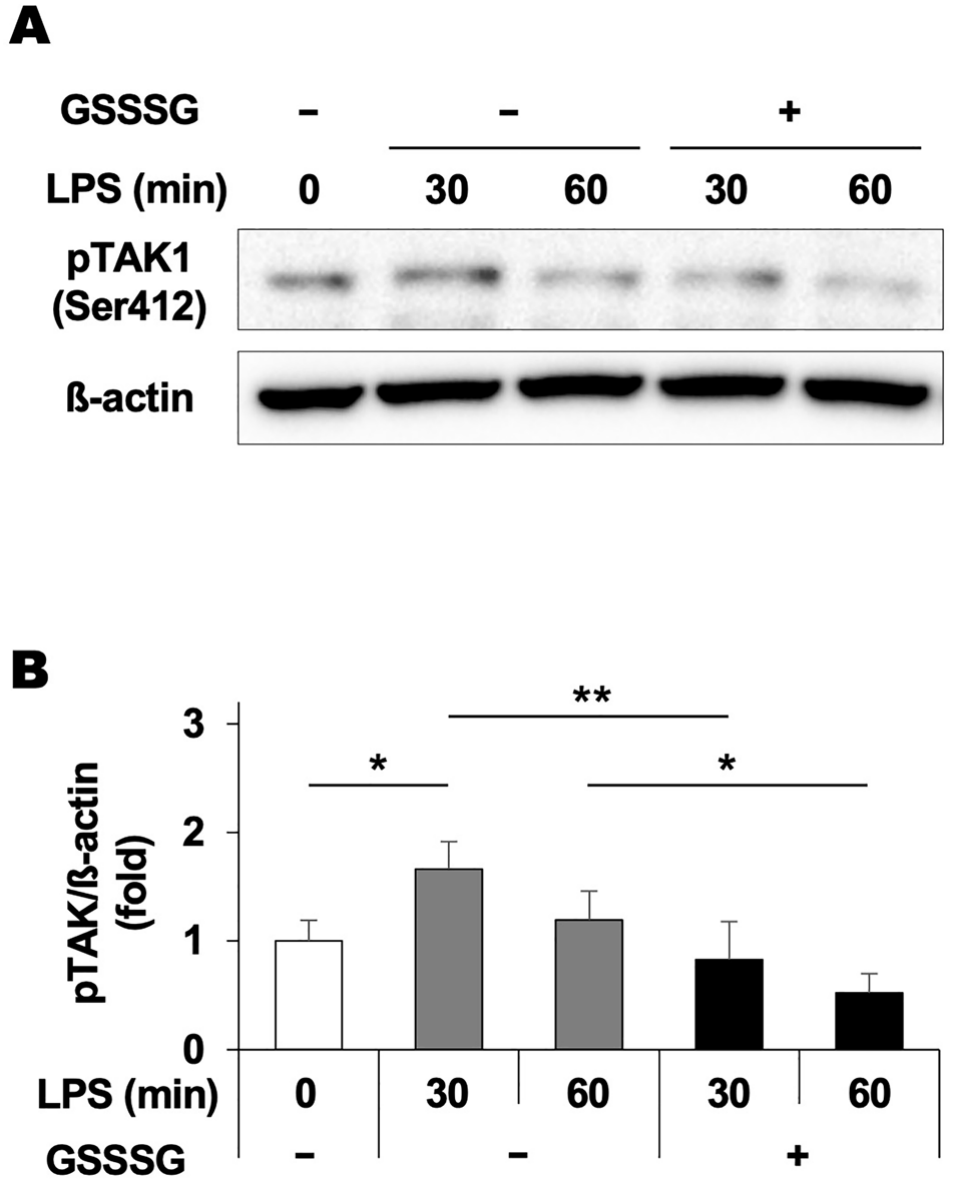
Inhibitory effects of GSSSG on LPS-induced TAK1 phosphorylation in BV-2 cells. (**A**) Immunoblots of BV-2 cell lysates with antibodies against phosphorylated TAK1 (Ser412) and β-actin as an internal control. BV-2 cells were pretreated with GSSSG (200 µM) for 1 h, followed by treatment with LPS (10 µg/mL) for 30 or 60 min. (**B**) Quantification of the immunoblot signals. The average was calculated from 4 independent experiments. Error bars represent the standard deviation of the mean. *P < 0.05, ^**^P < 0.01 (Tukey–Kramer test; *n* = 4).

The data from our preliminary test using arrays of antibodies against intracellular signaling molecules suggested that GSSSG administration inhibited TGF-β-activated kinase 1 (TAK1) phosphorylated at the serine 412 residue in BV-2 cells treated with both GSSSG and LPS compared to those treated with LPS alone (Supplementary Fig. S2). Thus, we investigated the time-course changes of TAK1 phosphorylation in BV-2 cells treated with GSSSG and/or LPS using immunoblotting. LPS caused a significant increase in TAK1 phosphorylation in BV-2 cells 30 min after administration (Fig. 6A,B and Supplementary Fig. S3). However, pretreatment with GSSSG for 1 h inhibited the LPS-induced enhancement of TAK1 phosphorylation at the time points examined (Fig. 6A,B and Supplementary Fig. S3).

## Discussion

Glial cells, including Müller cells and microglia, as well as retinal pigment epithelial cells are major sources of proinflammatory cytokines in retinas^41–43^. Excessive expression of these proteins exacerbates inflammation. Thus, it is important to properly control the expression level of proinflammatory cytokines upon exposure to harmful stimulants. In this study, we examined the impact of a glutathione polysulfide (GSSSG) on retinal inflammation and proinflammatory cytokine production in glial cells using LPS-induced in vitro and in vivo inflammation models.

Our previous in vitro study indicated that GSSSG exerted an inhibitory effect on proinflammatory gene expression in retinal pigment epithelial cells stimulated with LPS^33^. Consistently, the present study revealed that GSSSG could attenuate LPS-induced increase of proinflammatory cytokines at the mRNA and protein levels in glial cells, including Müller cells and microglia, which are key players responsible for regulation of the inflammatory state in the retina under pathological conditions^42,44^. Past studies indicated that attenuation of pathogen-associated molecular pattern-induced proinflammatory responses is coincident with inactivation of the NF-κB signaling pathway and hyperactivation of extracellular signal-regulated kinase (ERK) 1/2 following administration of *N*-acetyl-l-cysteine polysulfides or GSSSG in various kinds of established cells, including mouse macrophage-like and human retinal pigment epithelial cells^33,45^. Furthermore, ERK1/2 hyperactivation inhibited LPS-induced upregulation of proinflammatory genes without GSSSG^33^. Thus, it is possible that GSSSG exerts anti-inflammatory effects in glial cells via the same intracellular mechanisms. Additionally, this study revealed that GSSSG treatment inhibited phosphorylation of transforming growth factor (TGF)-β-activated kinase 1 (TAK1). TAK1 was originally identified as a potential mediator of TGF-β signal transduction and has been reported to be an upstream kinase of NF-κB^46,47^. LPS receptor toll-like receptor 4 (TLR4)-transducing signals are mediated by TAK1 activation^48,49^. Furthermore, TAK1 activates mitogen-activated protein kinases and NF-κB to trigger the production of proinflammatory cytokines^49^. Taken together with these previous findings, GSSSG-mediated deactivation of the TAK1-NF-кB pathway may contribute to the decreased proinflammatory cytokine production observed in this study.

This study is the first to demonstrate that GSSSG has the potential to attenuate LPS-induced inflammatory responses using in vivo experiments. GSSSG, but not its related molecule GSSG, known as a principal endogenous antioxidant, significantly inhibited proinflammatory gene upregulation in the retina of LPS-challenged rats. LPS administration leads to oxidative stress induced by reactive oxygen species (ROS). Furthermore, oxidative stress activates the NF-кB pathway to stimulate proinflammatory cytokine production^50,51^. GSSSG and GSSG both quench ROS through the redox reaction of thiol groups, although GSSSG is more effective^31^. However, elimination of oxidative stress seems not to be the mechanism underlying the GSSSG-mediated inhibition of LPS-induced proinflammatory gene upregulation. In a previous study, GSSG did not inhibit LPS-induced proinflammatory gene upregulation but rather stimulated it^33^. This is also supported with the finding that deficiency of nuclear factor-erythroid 2-related factor 2 (Nrf2), a master regulator of antioxidant defense responses including glutathione synthesis, had no effect on GSSSG-mediated inhibition of LPS-induced proinflammatory gene upregulation^33^. Thus, GSSSG would exert its anti-inflammatory activity by NF-кB inactivation and ERK hyperactivation through unidentified mechanisms other than antioxidation.

We found that intravitreal administration of GSSSG inhibited not only proinflammatory gene upregulation but also microglial accumulation in the retina of LPS-challenged rats. Resident microglia change the morphology from a ramified to larger and round shape upon activation with stimulants, as shown in Fig. 5B^52^. Activated microglia increase expression of proinflammatory cytokines including IL-1, IL-6, TNF-α, and Ccl2, which stimulate microglial proliferation^53–57^. Treatment with LPS led to activation of the TLR4-mediated NF-кB signaling pathway, implicated in proinflammatory cytokine upregulation, in microglia^58,59^. Our previous study indicated that GSSSG attenuated LPS-induced activation of the NF-кB signaling pathway through inhibition of p65 phosphorylation^33^. Taken together, a negative feedback effect of GSSSG on LPS-induced activation of the NF-кB signaling pathway and cytokine production may explain the mechanism underlying the GSSSG-mediated inhibition of microglial proliferation; GSSSG deactivates the NF-кB signaling pathway, which results in downregulation of proinflammatory gene expression in retinal cells including microglia. Consequently, decreased cytokine production leads to attenuation of LPS-induced microglial proliferation.

## Conclusion

In the present study, we examined the effects of a reactive sulfur species, GSSSG, on proinflammatory cytokine expression in glial cells in vitro and on retinal inflammation in rodents using LPS-induced inflammation models. Consequently, we found that GSSSG inhibits upregulation of proinflammatory cytokine expression in Müller cells and microglia, and secretion of these cytokines, as well as microglial accumulation in retinas. Furthermore, GSSSG-mediated deactivation of the TAK1-NF-кB signaling pathway may contribute to the cytokine downregulation.

Glial cells are major sources of proinflammatory cytokines in retinas and are implicated in inflammatory eye diseases. Thus, GSSSG-mediated regulation of glial inflammation could be effective in preventing the pathogenesis of such diseases.

## Supplementary Information

Supplementary Information.

## Data Availability

The datasets used and/or analyzed during the current study available from the corresponding author on reasonable request.
