# Supplementary material for: Glutathione trisulfide prevents lipopolysaccharide-induced retinal inflammation via inhibition of proinflammatory cytokine production in glial cells
Source: Sci Rep. 2023 Jul 17;13:11513. doi: 10.1038/s41598-023-38696-4 (PMC10352383; doi:10.1038/s41598-023-38696-4)
Supplement: Supplementary file 1 — Supplementary Information. [file 41598_2023_38696_MOESM1_ESM.doc]

**Glutathione Trisulfide Prevents Lipopolysaccharide-induced Retinal Inflammation via Inhibition of Proinflammatory Cytokine Production in Glial Cells**

**Hiroshi Tawarayama^1,2^, Kota Umeki^1^, Maki Inoue-Yanagimachi^1^, Naoki Takahashi^1^, Hirokazu Hasegawa^1^, Noriko Himori^1,3^, Satoru Tsuda^1^, Hiroshi Kunikata^1,2^, Takaaki Akaike^4^, Toru Nakazawa^1,2,5,6,7^**

^1^ Department of Ophthalmology, Tohoku University Graduate School of Medicine, Sendai 980-8574, Japan

^2^ Department of Retinal Disease Control, Tohoku University Graduate School of Medicine, Sendai 980-8574, Japan

^3^ Department of Aging Vision Healthcare, Tohoku University Graduate School of Biomedical Engineering, Sendai 980-8579, Japan

^4^ Department of Environmental Medicine and Molecular Toxicology, Tohoku University Graduate School of Medicine, Sendai 980-8575, Japan

^5^ Collaborative Program of Ophthalmic Drug Discovery, Tohoku University Graduate School of Medicine, Sendai 980-8574, Japan

^6^ Department of Advanced Ophthalmic Medicine, Tohoku University Graduate School of Medicine, Sendai 980-8574, Japan

^7^ Corresponding author

Corresponding author: Toru Nakazawa; Department of Ophthalmology, Tohoku University Graduate School of Medicine, 1-1 Seiryo-machi, Aoba-ku, Sendai 980-8574, Japan

Phone: +81-22-717-7294; Fax: +81-22-717-7293

E-mail: ntoru@oph.med.tohoku.ac.jp

**PROCEDURES**

***Antibody array.*** The Mouse Apoptosis Signaling Pathway Array C1 (RayBiotech Life, Peachtree Corners, GA, USA) was used to identify the signaling molecules implicated in GSSSG-mediated inhibition of proinflammatory cytokine upregulation in LPS-treated BV-2 cells. Briefly, BV-2 cells (6.6 × 10^5^ cells) were seeded in 60 mm cell culture dishes precoated with poly-D-lysine (Sigma-Aldrich, St. Louis, MO, USA). The next day, cells were pretreated with GSSSG (200 µM) for 60 min, and subsequently LPS was added to the cultures at the final concentration of 10 µg/mL in the presence of GSSSG. Cells were collected 60 min after LPS addition to the cultures, and then cell lysates were prepared using a cell lysis buffer. Protein concentration of cell lysates was determined using the Pierce BCA Protein Assay Kit (Thermo Fisher Scientific, Waltham, MA, USA). Prepared whole cell lysates (total proteins: 140 µg) were used for this experiment. Signal intensity was quantified with ImageJ software (NIH, Bethesda, MD, USA), and then the average and standard deviation were calculated from duplicate spots per every target molecule on the same membrane. The signal intensities obtained from the LPS and GSSSG-treated cells were expressed as relative values compared to those from the LPS-treated cells alone.

***Animals and histology.*** Wistar rats (8–10 weeks old) were intravitreally administered with 2 µL of GSSSG (total 60 nmol) or vehicle, and then sacrificed 48 h after administration. Dissected eyes were fixed with 4 % paraformaldehyde in PBS, and then cut into 15- and 30-µm sections, using a cryostat (Leica Biosystems Nussloch GmbH, Nussloch, Germany), for hematoxylin/eosin (HE) and immunohistochemical staining, respectively. HE staining was performed according to the routine procedure. Immunohistochemical staining was performed as described previously.^1^ Antibodies used for the purpose were shown in the Supplementary Table S2.

**Supplementary Table S1. List of the primer and probe mixtures used for quantitative RT-PCR.**

| Species | Genes | Supplier | Assay ID |
| --- | --- | --- | --- |
| mouse | Gapdh | Integrated DNA Technologies | Mm.PT.39a.1 |
| mouse | Il6 | Thermo Fisher Scientific | Mm.00446190_m1 |
| mouse | Il1ß | Thermo Fisher Scientific | Mm.00434228_m1 |
| mouse | Ccl2 | Thermo Fisher Scientific | Mm.00441242_m1 |
| mouse | Tnf-α | Integrated DNA Technologies | Mm.PT.58.12575861 |
| rat | Gapdh | Thermo Fisher Scientific | Rn.01775763_g1 |
| rat | Il6 | Thermo Fisher Scientific | Rn.01410330_m1 |
| rat | Il1ß | Thermo Fisher Scientific | Mm.00434228_m1 |
| rat | Ccl2 | Thermo Fisher Scientific | Rn.00580555_m1 |

**Supplementary Table S2. List of the antibodies used for immunochemical studies.**

| **Primary antibody** | | | |
| --- | --- | --- | --- |
| **Antigen** | **Supplier** | **Catalog #** | **Dilusion** |
| CRALBP | abcam | ab15051 | 1:1,000 |
| GFAP | Dako | Z0334 | 1:2,000 |
| phospho-TAK1(Ser412) | Merck-Millipore | 06-1425 | 1:1,000 |
| ß-actin | Sigma-Aldrich | A5316 | 1:10,000 |

| **Secondary antibody** | | | | |
| --- | --- | --- | --- | --- |
| **Fluorophore** | **Antigen** | **Supplier** | **Catalog #** | **Dilution** |
| AlexaFluor488 | mouse IgG | Jackson ImmunoResearch | 715-545-150 | 1:1,000 |
| Cy3 | rabbit IgG | Jackson ImmunoResearch | 711-165-152 | 1:1,000 |
| HRP | mouse IgG | Jackson ImmunoResearch | 715-035-150 | 1:100,000 |
| HRP | rabbit IgG | Jackson ImmunoResearch | 711-035-152 | 1:100,000 |


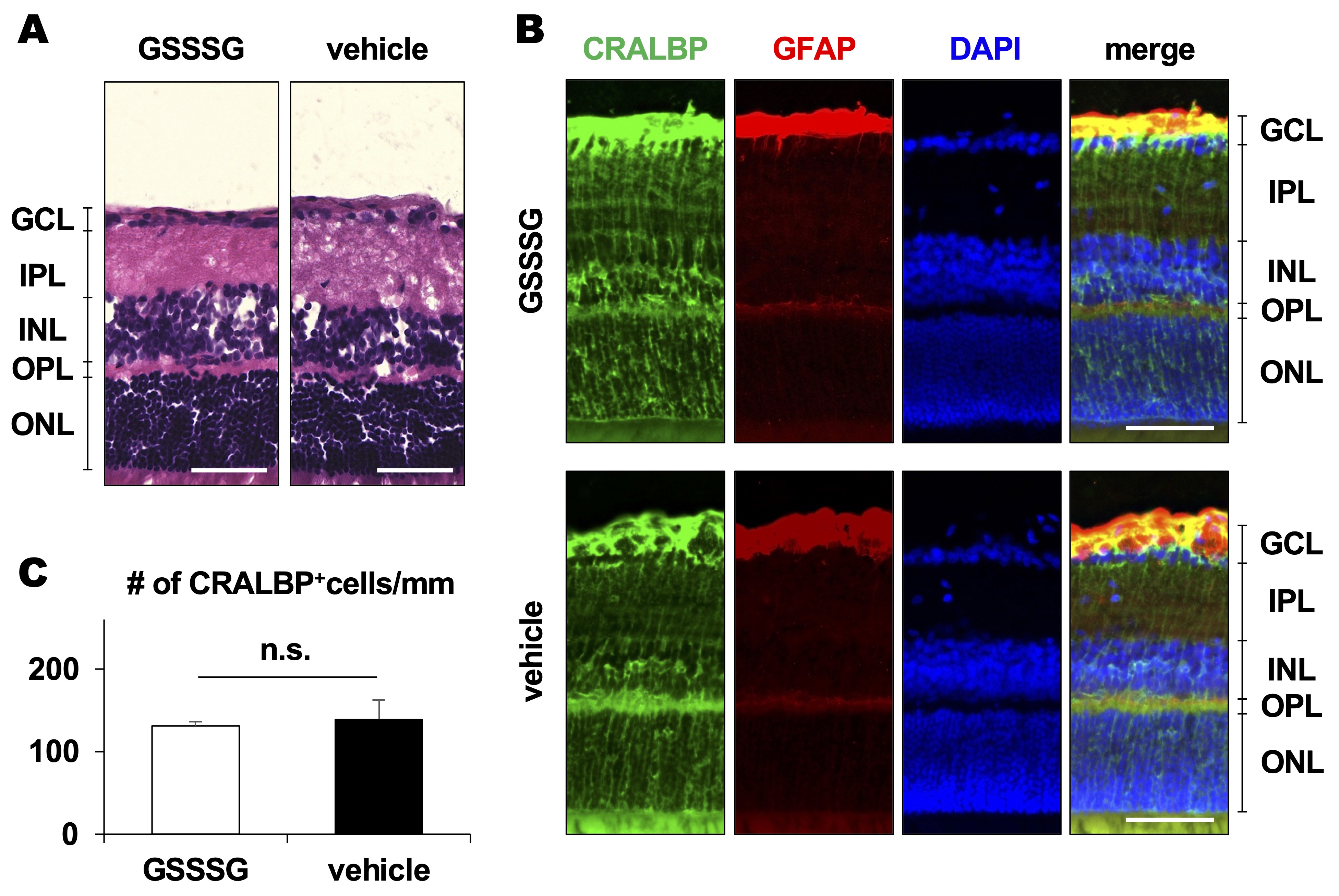
**Figures**

**Supplementary Figure S1. Effects of GSSSG on the retinal structure and expression of glial cell markers.** (**A**) Hematoxylin and eosin (HE) staining of retinal sections prepared from Wistar rats which received intravitreal injection of GSSSG (60 nmol) or vehicle. (**B**) Immunohistochemical analysis for glial markers, CRALBP (cellular retinaldehyde-binding protein) and GFAP (glial fibrillary acidic protein) on the retinal sections. No drastic changes were observed in the retinal layer structure and expression patterns of a Müller cell marker CRALBP and glial marker GFAP. (**C**) Quantification of CRALBP-immunoreactive Müller cells in the inner nuclear layer of the retina. The number of Müller cells was counted in three randomly selected 100-µm regions on the same section from each retina, and the average number per mm was calculated. The grand mean was derived from the averages of three (for GSSSG) or four (for vehicle) independent retinas. n.s.: not significant (Welch's t-test). GCL: granule cell layer, IPL: inner plexiform layer, INL: inner nuclear layer, OPL: outer plexiform layer, ONL: outer nuclear layer. Scale bar: 50 µm.

**
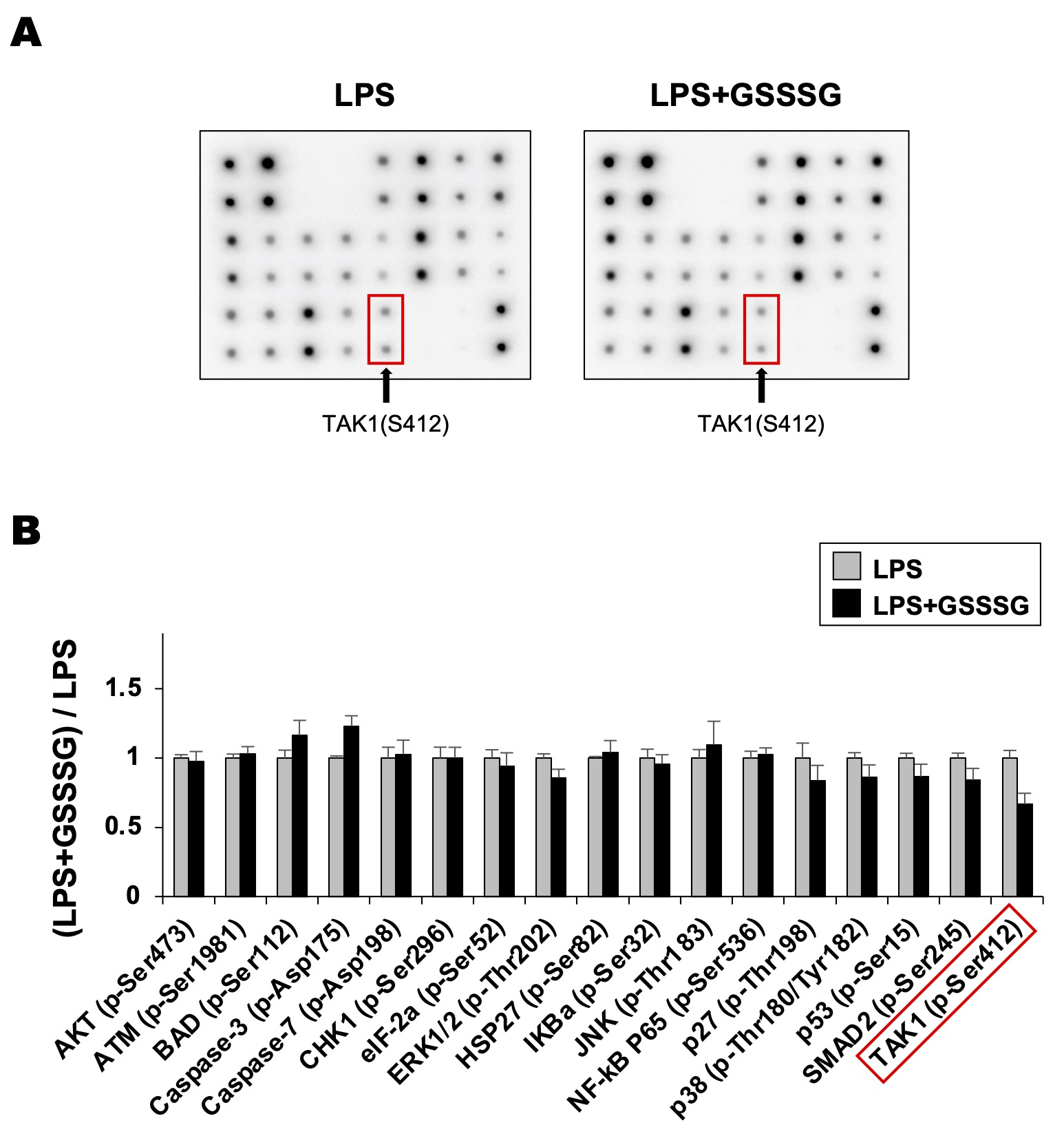
**

**Supplementary Figure S2. Effects of GSSSG on the activation of the intracellular signaling molecules involved in inflammation, survival, and apoptosis.** (**A**) Dot-blot assay using antibody arrays with immobilized antibodies recognizing phosphorylated proteins. (**B**) Quantification of dot blot signals in (**A**). The signals from cell lysates of LPS/GSSSG-treated cells were expressed as a percentage relative to those of LPS-treated cells. The error bars represent the standard deviation of the mean. The quantification data suggested deactivation of TAK1 (TGF-ß-activated kinase 1) upon GSSSG treatment.


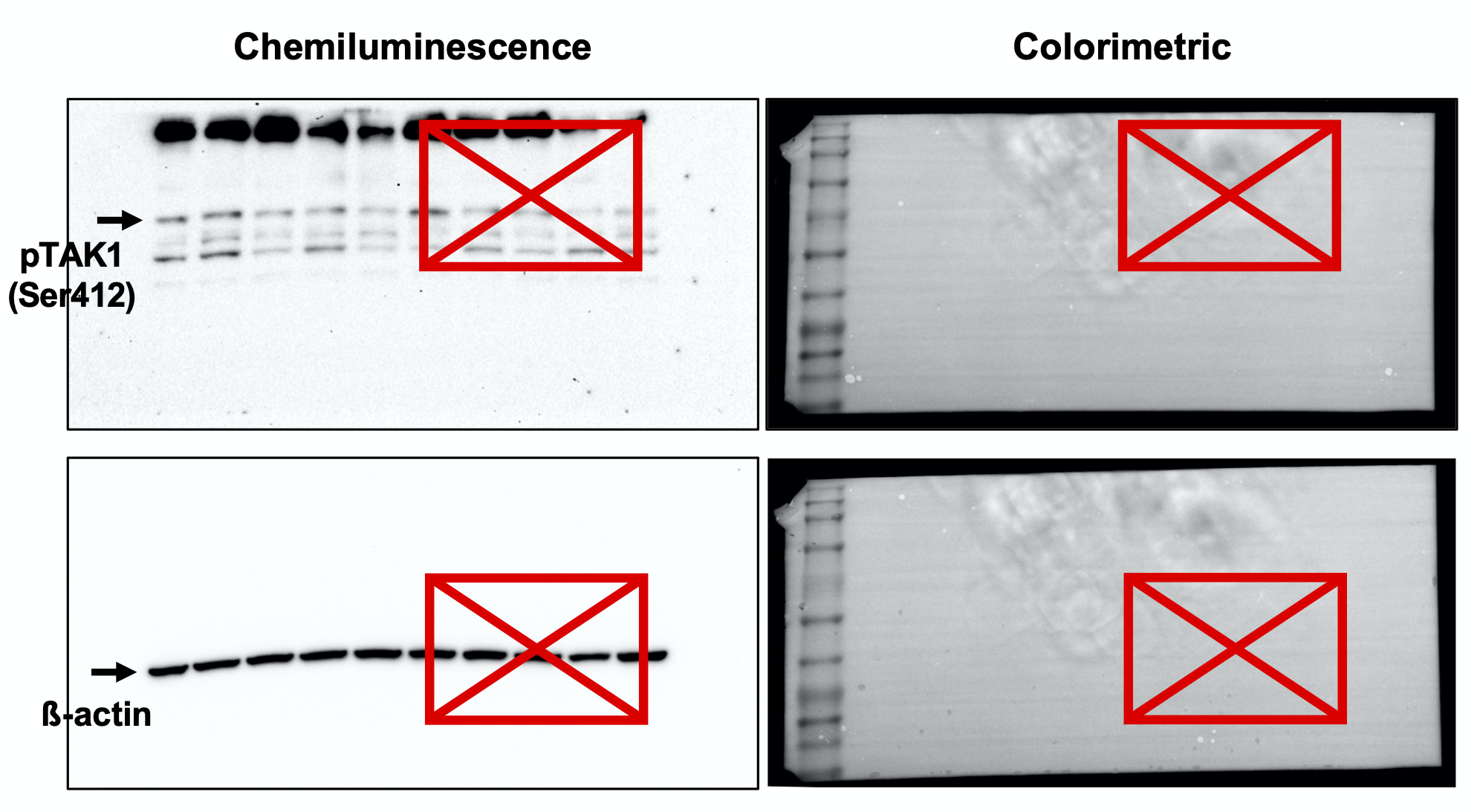


**Supplementary Figure S3. Immunoblots of cell lysates of BV-2 cells treated with LPS and GSSSG.** The uncroped images of the immunoblot data displayed in Fig. 6A are presented here. The left and right columns respectively show colorimetric and chemiluminescence images of membranes that have been immunoblotted with antibodies against phosphorylated TAK1 (upper images) and ß-actin (lower images).

**References**

1. Tawarayama, H., Feng, Q., Murayama, N., Suzuki, N. & Nakazawa, T. Cyclin-dependent kinase inhibitor 2B mediates excitotoxicity-induced death of retinal ganglion cells*. Investig. Ophthalmol. Vis. Sc*i**.** 60, 4479–4488 (2019).
